# Experiences, Challenges, and Coping Strategies of Frontline Healthcare Providers in Response to the COVID-19 Pandemic in Kelantan, Malaysia

**DOI:** 10.3389/fmed.2022.861052

**Published:** 2022-05-19

**Authors:** Ruhana Che Yusof, Mohd Noor Norhayati, Yacob Mohd Azman

**Affiliations:** ^1^Department of Family Medicine, School of Medical Sciences, Universiti Sains Malaysia, Kubang Kerian, Malaysia; ^2^Medical Practice Division, Ministry of Health, Federal Government Administrative Centre, Putrajaya, Malaysia

**Keywords:** COVID-19, healthcare providers, pandemic, psychological, qualitative

## Abstract

**Introduction:**

In the fight against the COVID-19 pandemic, frontline healthcare providers who are engaged in the direct diagnosis, treatment, and care of patients face a high risk of infection and inadequate protection from contamination, overwork, frustration, and exhaustion. These impose significant psychological and mental health concerns for frontline healthcare providers.

**Objectives:**

This study aimed to explore the experiences and challenges faced and coping strategies adopted by frontline healthcare providers in response to the COVID-19 pandemic in Kelantan, Malaysia.

**Methodology:**

This phenomenological approach to qualitative study used a telephone-based in-depth interview that followed a semistructured interview guide. The number of frontline healthcare providers was based on saturation theory. All the participants recruited fulfilled the inclusion and exclusion criteria from May to July 2020 in Raja Perempuan Zainab II Hospital. All interviews were audio recorded and transcribed verbatim. Thematic data analysis using NVIVO version 10 was performed.

**Result:**

The 10 respondents involved in this study consisted of doctors, medical assistants, and nurses. The findings were divided into four main themes: invaluable experiences during the pandemic, challenges, coping strategies, and future expectations. The providers responded well in facing the disease even though they felt psychologically disturbed at the initial phase of the COVID-19 pandemic.

**Conclusion:**

Healthcare providers perceived themselves as being more resilient and less vulnerable to psychological impacts than they were before the pandemic.

## Introduction

In early 2020, the world was assailed by a new disease known as coronavirus disease 2019 (COVID-19) (1). This deadly pandemic has substantially impacted the economies, health, and social life of people worldwide.

The first wave of COVID-19 in Malaysia, 22 confirmed cases, was detected between January 2020 and mid-February 2020. The second wave of COVID-19 was related to a mass religious gathering in Kuala Lumpur, with a three-digit number of cases per day involving different states that shocked the whole country (2). This drastic increase in the number of infections caused changes in the management of health facilities, especially in hospitals managing COVID-19 patients. As a result, some healthcare providers, such as medical officers, medical assistants and nurses, have been assigned to frontline teams, and other healthcare providers had to take over the tasks of those so deployed. Consequently, management has to reschedule the working hours and shuffle the duties of health staff to ensure that their services will not be interrupted while they battle COVID-19.

Even though some healthcare providers have experienced the epidemic of severe acute respiratory syndrome and the Middle East respiratory syndrome, the vigilance of COVID-19 does not prevent healthcare providers from being affected, and it is proven that COVID-19 is the most severe and troublesome of all diseases (3), causing the highest prevalence of mortality and burden on hospital in-patient facilities, as well as personal protective equipment (PPE) shortage issues (4). In a way to respond to the COVID-19 pandemic, the capacity of the healthcare providers has been enhanced to accommodate healthcare demands (5–7). In Philippines, the healthcare system has reached its full capacity and the government was urged to prioritize the need for healthcare workers who experienced physical exhaustion and emotional distress (8).

Healthcare providers were observed experiencing psychological effects such as acute stress, depression, anxiety (9), and posttraumatic stress disorder (10). Exposure directly to COVID-19 patients (11), isolation and quarantine, deployment to epidemic areas (12), and increased working hours and workloads (13) are the common factors related to this psychological distress. Providers were also anxious about their safety and the risk they might transmit the disease to their family members (14). However, they need to be strong in performing their social and professional services in fighting against COVID-19 (14). They were reported to have increased resilience in facing psychological disturbances such as fear, stress, anxiety, and depression during the pandemic (15).

The experiences gained by healthcare providers working with COVID-19 have been invaluable. They are gaining more treatment knowledge and skills, regaining self-confidence in facing the pandemic, and learning how to live and getting used to living a new life with the disease (16). They have been challenged by inadequate supplies of equipment like PPE, which has led them to reuse it and improvise new versions of it (17); the increase in patients has caused a shortage of health staff (18) and made the available staff work more hours under increased workloads; they fight with their fear of being infected (19) and transmitting the disease to their colleagues and family members; however, they have successfully coped with and adapted to the situation and continue their work and life as usual in the “new normal.” Undeniably, government policies and management by their leaders, their colleagues, and their family members contribute to supporting them as they manage their psychological distress from battling the pandemic.

Coping strategies by healthcare providers may come from sharing emotions through peer support or problems, such as training in and practice of proper techniques and skills, or on religious and/or spiritual matters (20). Moreover, engaging in stress reduction activities such as exercise or physical activities, talk therapy, and virtual support teams may help them minimize their psychological disturbance and cope with the situation (9).

The experiences, challenges, and coping strategies in response to COVID-19 differ among healthcare providers. Providers may be influenced by many factors, such as personal experiences, management's role, and the available resources and support systems provided for them. Therefore, this study aims to explore the experiences and challenges faced by frontline healthcare providers and the coping strategies they have adopted in response to the pandemic in Kelantan using a qualitative study approach.

## Methods

### Setting and Participants

A descriptive phenomenological approach was followed in this qualitative study. This approach was focused to understand the core lived experiences of frontline healthcare providers during the COVID-19 pandemic. In-depth interviews were conducted among healthcare providers in intensive care units in a state tertiary hospital that managed suspected and confirmed COVID-19 cases in Kelantan, Malaysia. Doctors, nurses, and medical assistants were included. Those diagnosed as having psychiatric illnesses were excluded. Purposive sampling was applied. The number of frontline healthcare providers interviewed was based on saturation theory; the interview ends when until no new themes emerged from the respondents or until no further information could be obtained (21). In phenomenological studies, a sample between 5 to 25 is considered adequate (22).

### Data Collection

The most common method for conducting interviews is face-to-face, but studies have revealed no significant difference between face-to-face and telephone interviewing in qualitative research (23, 24), so interviews were conducted by telephone. Qualitative telephone interviews can limit emotional distress because of the comfort experienced through virtual communication (25). Front liners are at increased risk of developing stress due to cumulative exposure to COVID-19 in the hospitals managing the pandemic. A semistructured interview guide was utilized and covered three areas: (i) work-related and personal experience, (ii) challenges in management and (iii) methods for coping.

Eligible respondents were invited through phone calls. For each respondent who agreed to be interviewed, the appointment date and time were set. The interview was conducted at the completion of the respondent's shift and audio recorded. During the appointment, the researcher introduced herself to the respondents and explained her interest in understanding their experiences. The audio recording was anonymous, and the respondents were assured that their information would be kept confidential. The respondents who agreed to participate in the research provided verbal consent and responded to a virtual consent form sent through WhatsApp. Each interview lasted approximately 45–60 min. A summary and a reflection of the salient points of the interview were documented after the interview. The entire conversation was transcribed verbatim in the language of the respondents.

### Data Analysis

The verbatim transcripts were managed using computer-aided qualitative data analysis software, namely NVIVO version 10 (QSR International Pty Ltd., 2012). The analysis took place concurrently with the data collection and continued until the saturation point was reached. The respondents were identified by identification numbers to maintain the confidentiality of the findings.

Thematic data analysis, a process of encoding information, was done in six phases (26): (i) Familiarization with the data: the transcripts were read and re-read to familiarize the researchers with the information and make sure they understood its overall meaning. (ii) Generation of initial codes: the entire dataset was coded, and labels were generated for important information. The preliminary coding framework was based on the synthesis of initial concepts identified in previous studies. As the process continued, emerging codes were recorded and compared with the initial codes. (iii) Categorization of themes: the codes were combined to create subthemes (subcategories) and then condensed to generate themes (categories). (iv) Review of themes: the themes were reviewed and split, combined or discarded as needed. (v) Defining and naming of themes: Meanings were formulated for the categories or themes. (vi) Production of the report: the analytic narrative and the extracts were woven together, and vivid examples were chosen to demonstrate the essence of a point.

In a qualitative study, rigor is attained by implementing reliability and validity during the inquiry (27). Reliability is measured by the consistency of the research. In this study, the researcher checked the transcripts by listening to the audio recordings to ensure errors were not introduced during transcription and coding. During the coding process, data were compared continuously with codes to ensure that no changes occurred to the meaning of the codes. The coding for the same text was cross-checked and verified by a co-researcher.

Validation is a judgment of the trustworthiness of a piece of research. Creswell has proposed eight validation strategies for qualitative research (28) and three of which were used in the current study. First, through the member checking process, the polished content of the transcripts and the quotations cited were read to the respondents. This was done by phone calls after the interviews were transcribed and the themes constructed. Second, a rich, thick description was used to provide many perspectives about a theme so that the results became richer and more realistic. Third, negative, discrepant, or contrary information was presented to add credibility to the account.

## Results

Ten healthcare providers from intensive care units (ICUs) were interviewed between May and July 2020. Six were female, four male. Two were medical officers, two medical assistants, and six nurses. Four were transferred staff from the surgery department into the ICU for managing COVID-19 patients and patients under COVID-19 investigation. Five were directly involved with COVID-19 patients; the others acted as backups, runners, floaters, and handlers of machines like ventilators in the ICU and operation theater. Eight of them were married and living with family members; two were single, one living with parents and the other living alone.

The exploration of the experiences challenges faced, and coping strategies adopted by the frontline healthcare providers, as well as their hopes for the future, are summarized in Table 1 with the themes and subthemes that emerged from the data analysis.

**Table 1:** Thematic analysis.

**Themes**	**Subthemes**	**Initial context**
Invaluable experiences during pandemic	1. Hospital management	a. Infrastructure management b. Staff management
	2. COVID-19 patients management	a. Death b. Handling of high-risk cases c. Health staff infected with COVID-19 d. Health staff quarantine
	3. COVID-19 screening activities	a. Swab test screening
	4. Feelings during pandemic	a. Fear b. Fear of COVID-19 transmission to family members c. Ability to manage the situation d. Sadness e. Stress
Challenges faced by healthcare providers	1. Battling with inner psychological disturbances	a. Community behaviors at large b. Immediate family health concerns c. Interpersonal relationships d. Psychosomatic reactions e. Health concerns for self
	2. Provision of PPE	a. Shortage of PPE b. Donning and doffing
	3. Administrative and work adjustment	a. Bureaucratic issues with bonus allocation b. Shuffling of staff c. Unfamiliar work environment
	4. Screening and COVID-19 infection symptoms	a. Access to screening b. Have I contracted COVID-19 infection?
	5. Management of confirmed and probable cases	a. At high risk b. Presumed positive till proven otherwise
Coping strategies	1. Clinical governance	a. Rearrangement of schedules b. Teamwork, management, and support
	2. Knowledge and adherence to standard operating procedures (SOP)	a. Knowledge of COVID-19 b. Practice of good control measures c. Prevention of transmission to others d. Precautions for self
	3. Physical resources	a. Availability of PPE b. Control of PPE use c. Prioritization of staff for PPE use d. Initiatives for producing PPE for self
	4. Spiritual and mental strength	a. Good mindset and motivation b. Inner self-adjustment c. Religious practices d. Sincere and patient acceptance of responsibility
	5. Preparation for worse situation	a. If cases increase again b. Preparation if cases increase again
Future expectations	1. Hope 2. Lessons learned 3. Recommendations	

### Theme 1: Invaluable Experiences During the Pandemic

The first theme describes the experiences that the healthcare providers had during the management and handling of COVID-19 cases. The experiences were related to management, screening, and feelings. The findings were subcategorized into hospital management, COVID-19 patient management, screening activities, and feelings during the pandemic.

#### Subtheme 1.1: Hospital Management

The hospital has created standard operating procedures for managing COVID-19 cases, which were separated from other cases as part of infrastructure management. The ICU, with all necessary equipment and the operating room for patients, was prepared separately and divided into two areas: a “dirty area” and a “clean area.” The “dirty area” was an area where they treated the patients, and providers were obligated to wear full PPE to enter the area; the “clean area” was where they did the necessary documentation of the patients and did not need to wear full PPE. There was a team to handle the receiving of patients into the ICU. They needed to be informed about the admission of each patient so they could clear all the pathways and prepare themselves to receive him or her. The patient was transferred using an “isopod,” special equipment to reduce the exposure to other persons during the process. The equipment was handled by well-trained staff. The hospital also decided to transfer non-COVID-19 patients to another hospital to minimize other patients' exposure to the disease.

There was a reshuffling of staff like medical officers, medical assistants, and nurses from other departments into the ICU and changes in shift schedules for staff management. Medical officers and medical assistants worked in three shifts in every 24 h, seven to 8 h per day shift and 10 h per night shift; nurses worked in two shifts of 12 h each. They alternated two working days with two off days to maintain their physical and psychological health. Each patient was assigned one medical officer and two nurses, an inside nurse on duty in the “dirty area” and an outside nurse in the “clean area.”

#### Subtheme 1.2: COVID-19 Patient Management

The COVID-19 patient management subtheme describes the staff's experiences inpatient death, handling high-risk cases and staff infected with COVID-19, and staff quarantine. There was a case where a severe patient under investigation (PUI) for COVID-19 died two or 3 h after admission to the ICU. The ICU staff prepared the deceased for transfer out to the mortuary, and the mortuary staff performed the other related procedures.

In the early phase of the pandemic, the healthcare providers in the study setting did not handle many confirmed cases of COVID-19. The cases they did handle were PUIs with severe acute respiratory infections (SARIs) or influenza-like illnesses (ILIs). The related cases were treated as COVID-19 cases until proven otherwise. The frontline healthcare providers prepared themselves with new knowledge about COVID-19, and they were alerted to and followed the guidelines, standard operating procedures, and control measures related to this disease, including the ways they handled ICU equipment like ventilators.

Frontline healthcare providers are at the highest risk of COVID-19 infection. When the health staff is infected, they may expose patients, colleagues, and family members to it. The healthcare service may be interrupted if transmission occurs among health staff. To prevent this, providers need to be screened and isolate themselves when symptoms appear. They need to inform their close contacts to do a swab screening and take necessary precautions. One medical officer was infected after being in close contact with an infection-confirmed colleague. He transmitted the infection to his family members, but he felt relieved when the hospital management accommodated them together in the same ward with a good hospitality and support system.

Another situation occurred when the health staff needed to be quarantined after being in close contact with an infected colleague. If the staff had symptoms of infection, they needed to do a swab screening and isolate themselves. They were given three days of medical leave and needed to do swab screenings twice. If the second swab screening result was negative, they could return to work as usual. If the result of the swab screening was positive, they were admitted to a hospital until they recovered. After discharge from the hospital, they were given another 14 days of medical leave for home quarantine before coming back to work. A total of 34 days of leave were provided for medical officers who had been, including hospital admission and home quarantine.

#### Subtheme 1.3: COVID-19 Screening Activities

Due to limited human and material resources, such as lack of staff and an overflow of applications, COVID-19 swab test screenings were provided only to frontline healthcare providers who had symptoms or were in close contact with infected persons. Health staff who had no symptoms were not prioritized for a swab test screening. However, all health staff had daily temperature screenings and needed to be alert for the appearance of symptoms.

#### Subtheme 1.4: Feelings During the Pandemic

The COVID-19 pandemic has triggered a variety of feelings among frontline healthcare providers. They fear transmitting COVID-19 to family members; they feel sad, stressed, and barely able to manage their mental health. Most health staff felt fear and insecurity in their ability to protect themselves due to a lack of PPE during the early pandemic phase. This fear was also triggered due to lack of knowledge about COVID-19, changes in their working places and environments, unsureness whether they were dealing with COVID-19, SARI, or ILI, and exposure to infected colleagues. The situation made one of the health staff want to apply for unpaid leave, retire early, or even quit the job if given a choice.

Some health staff also fear COVID-19 transmission to family members even though they have taken precautions such as bathing before returning home and keeping a distance from family members. Most of them have high-risk family members who live with them, such as children, elderly parents, and parents with chronic diseases like diabetes. However, some other health staff were not afraid and felt confident that their practices and precautions would prevent the spread of the disease to their family members.

One of the respondents felt no stress because he was not involved directly with COVID-19 patients. He believed that he had not been exposed because the management of the hospital had managed the COVID-19 cases well. Another respondent, a medical officer, also admitted that he did not feel stressed since there were only a few confirmed cases in the hospital, and the working hours were well scheduled. In other words, the workload was lighter during that time.

“*Only a few cases of positive COVID-19 were admitted to our ICU. There were more cases of PUI and SARI. The shift roster made us feel less stressed. Everyone works seven to 8 h per shift. Then we will have a few days off. So, we feel less stress.”* (MO06)

This medical officer felt sympathy toward his nurse colleagues who had been exposed to COVID-19 longer while nursing patients with 12 h shifts compared to his 8 h shifts. The nurses also felt sad that they had to work in dangerous conditions with a lack of protective equipment. It became worse for nurses with no ICU ward experience who had been shuffled in from other departments.

“*At first, I was shocked because I was assigned to the ICU. I did not have any experience in the ICU. I had ward duty experience, but the ICU is different. It is an intensive care unit. Must handle machines. So, I was shocked. I did not know anything about the ICU. I felt fear and sadness. I had no problem working there, but I was afraid due to the lack of PPE. I had to wear a plastic garbage bag as a replacement for PPE.”* (N10)

The nurses had more stress than did the medical officers because of the stress of having inadequate PPE while attending to COVID-19 patients. Meanwhile, the medical officers felt less stress due to more manpower from other departments that could cover the ICU. Thus, the workload of medical officers for managing the COVID-19 ICU patients was lower.

### Theme 2: Challenges Faced by Healthcare Providers

The second theme is challenges faced by healthcare providers, which consisted of five subthemes: (i) battling with inner psychological disturbances, (ii) provision of PPE, (iii) administrative and work adjustment, (iv) screening and symptoms of COVID infection, and (v) management of confirmed and probable cases.

#### Subtheme 2.1: Battling With Inner Psychological Disturbances

The frontline healthcare providers needed to batt inner psychological disturbances. For instance, they felt frustrated with the behavior of those in the community who disobeyed the order to stay at home. Even though the government had imposed a movement control order (MCO) had been imposed in that area to reduce exposure to and transmission of COVID-19, some stubborn groups of people still left their houses without any important business.

Four respondents were concerned about their family members' health. They were afraid to bring home the disease since most of them live with members of high-risk groups like older parents and young children. When they returned home, some of them kept their distance and isolated themselves from their family members.

Workers during the pandemic became more sensitive to their inner psyches, which influenced their relationships with their colleagues. One of the respondents complained that her higher-level colleague was offended by her reprimand about the right way to handle the documentation of COVID-19 patients.

“*I had reprimanded a senior medical officer about [the way he handled] a patient's physical medical record in PPE after seeing a patient. [Since] then, it has become a problem. He does not want to listen to me even though I have used a nice way to talk, but my tone of voice is high.”* (N03)

Another respondent complained that even though he had initially been content working in the new shift, he had started to feel dissatisfied after being influenced by his colleagues, who repeatedly expressed dissatisfaction about working more hours without bonus after the change in their working shifts. Meanwhile, a nurse complained that her sleep was being disturbed during her on-call shift.

“*During the night on call, I cannot sleep because I heard a phone ringing even when there was no call.”* (N01)

Healthcare providers cannot escape from their own health concerns. In the early phase of the COVID-19 pandemic, they felt increased anxiety about their exposure to infection, whether from patients or colleagues. If given a choice, they would have chosen to take leave during that time. As their duty and responsibility, however, they did the work with extra precautions and followed the standard operating procedures and protocol of COVID-19 control measures.

#### Subtheme 2.2: Provision of PPE

In the first week of COVID-19 cases, there was a worldwide shortage of PPE, including in the study setting, which caused management to restrict usage of PPE to essential services that directly involved COVID-19 cases. They rescheduled the working shift to minimize the use of PPE and at the same time minimized the health staff to prevent exposure to COVID-19. However, health staff also worked together to find solutions to the shortage of PPE.

Donning (putting on) and doffing (taking off) of PPE is an essential procedure for all healthcare providers directly in contact with COVID-19 patients. A complete set of PPEs consisted of a gown, a coverall, a glove, a respirator, and an eye protector. The gown's raw material, the coverall, is made with a spun-bond or melt-blown non-woven synthetic, typically polypropylene, polyester, or something similar. This PPE makes the users uncomfortable, overheating them and making it difficult for them to work, especially when they are worn for a long time.

“*I felt suffocated wearing the PPE. It was hot. Also, it was difficult to wear double gloves and difficult to move around. So, it was a bit difficult to handle the PPE.”* (MO09)

#### Subtheme 2.3: Administrative and Work Adjustment

The third challenge faced by healthcare providers was bureaucratic issues with bonuses. The Ministry of Health announced that the health staff could claim bonus if they were involved in COVID-19 cases. However, one of the nurses claimed that she should get a bonus because she had been involved in COVID-19 cases, but the bureaucrats claimed that she was not eligible to receive the bonus because the patients she attended were only PUI.

“*I heard the sister nurse say those who were assigned to ICU Intan could not make a claim. It is okay if you cannot claim. It does not matter. But we want our emotions in stable condition.”* (N08)

During the escalation of COVID-19 cases, the hospital management decided to shuffle the staff to fulfill the demand for frontline staff. In the surgery department, they had closed several operation theaters. They sent their teams to different locations, such as wards, ICUs, and other hospitals, to cover staff shortages. It was very challenging for the staff who entered new departments.

“*[That was] the first experience in my life, the first in 23 years of working, that was the first time I was assigned to an ICU ward. As I qualified as a nurse, I had never been exposed to the ward. Ward management and ICU management are different. I do not know how to make a report. Zero-knowledge. I felt like a five-year-old child who starts learning all those things. But the majority who were assigned there were stressed. I cannot imagine talking to the sister or matron, who said that was the responsibility we had to perform.”* (N08)

“*I followed the ICU staff nurse. I just followed her. I followed her attending the patient, performing procedures, and handling and operating machines that I had never handled as a scrub nurse. So, I had to learn many things. I was stressed at first but adapted over time.”* (N10)

Workloads were increased, especially for nurses, who had to change their work schedule from 8 h per day to 12 h per day. However, they were given two off days after two working days. This change also benefitted them in terms of less exposure to COVID-19 infection.

Since they worked in new departments, they also needed to learn and adapt to the unfamiliar environment. They always needed to wear uncomfortable protective equipment, learn to handle new equipment like ventilator machines, learn new techniques and procedures, and even face patients' deaths. All these situations challenged their physical and mental health.

#### Subtheme 2.4: Screening and COVID Infection Symptoms

High laboratory test costs and limited resources for swab test screening due to the increasing number of COVID-19 cases have influenced health staff members' access to COVID-19 screening. In the early phase of the pandemic, the government only used real-time reverse transcription-polymerase chain reaction (rRT-PCR) tests as the gold standard COVID-19 diagnostic test. This test requires more time for analysis before the result can be reported, and the machine for the test is not available in all health facilities. Therefore, only selected health staff is eligible to do the test at the hospital. The conditions for health staff eligible for the swab test screening for rRT-PCR tests were those with close contact with COVID-19 persons and had symptoms like fever, sore throat, cough, or difficulty breathing. If they fulfilled the conditions, they could undergo swab test screening at the hospital.

In the study setting, there were a few health staff members who had been in close contact with COVID-19 infection. They were required to self-quarantine and went for screening if they developed symptoms. However, some had symptoms, but they did not do the screening since they had not had contact with COVID-19 patients, and they felt well. They just monitored their health.

#### Subtheme 2.5: Management of Confirmed and Probable Cases

Management of a high-risk COVID-19 patient is different from the usual case and takes longer than in other cases. Staff members felt that managing patients was complicated since they had to prepare well before receiving the patient and needed to clean the room after the patient left.

“*COVID on-call—it was complicated. To finish a case, for example, from 10 pm, we started the case with a 2 h operation, supposedly finished at midnight for a usual case. But for COVID cases, we needed more time to transfer the patient, doffing, and clearing the operating room.”* (N01)

They also needed to presume that all PUI, SARI, and ILI patients were COVID-19 positive until proven otherwise. They needed to clear and sanitize all the routes for transferring patients to the ward, treat all waste as COVID-19 biohazard, correctly don and doff PPE, and always sanitize all the equipment and the patients' rooms, and clean themselves well to protect themselves from the infection.

### Theme 3: Coping Strategies

Coping strategies for healthcare providers to manage their psyches during the COVID-19 pandemic are divided into five sections: (i) clinical governance, (ii) knowledge and adherence to standard operating procedures, (iii) physical resources, (iv) spiritual and mental strength, and (v) preparation for worse situations.

#### Subtheme 3.1: Clinical Governance

To have smooth clinical governance, the management decided to rearrange the working schedule and empower teamwork and support among healthcare providers. They called back the housemen from other departments to cover the medical officers in the ICU, called all medical assistants to assist screening teams, and reshuffled the nurses to cover the COVID wards and ICUs.

Good teamwork, management, and support systems have made these frontline healthcare providers feel at ease in doing their duties. They helped and supported each other in their battle against this pandemic. The management also took part in the staff's welfare through physical and psychological care and support.

#### Subtheme 3.2: Knowledge and Adherence to Standard Operating Procedures (SOPs)

Information and knowledge about COVID-19 have helped these frontline healthcare providers cope with this new situation quickly and overcome their worries while performing their duties.

“*So, I feel confident with my knowledge about COVID. I return home safely in a safe condition. I did not bring home the COVID virus.”* (MA05)

Staff members were constantly reminded to adhere to standard operating procedures and practice good control measures such as the proper wearing of protective equipment, hand hygiene and sanitization, and maintenance of distance between each other. They also tried to have less exposure to their patients and took extra precautions by bathing before going back home. They also practiced these safety measures in the community and advised the public to practice them.

To prevent transmission to others, they tried to go home safely. They bathed at work and again in their houses before meeting with their families. Some of them practiced social distancing with their families and did separate laundry to make sure the virus was not transmitted to their family members. Providers who had symptoms isolated themselves from their families in different rooms or did not otherwise meet directly with them.

For precautionary measures for themselves, the management practiced a rotating system where nurses had to rotate out after every 2 h of attending to patients to make sure that they had less exposure to the disease. They also maintained social distance with their colleagues even during unit meetings in the on-call room or the prayer room. If they had close contact with COVID-19-positive colleagues and had symptoms, they needed to do a swab test screening and quarantine themselves until the result came out.

#### Subtheme 3.3: Physical Resources

In coping strategies, physical resources, such as the availability of PPE, are an important aspect of providing good mental health while working during pandemics. Staffers worked with peace of mind when they felt safe and well protected.

“*With an adequate PPE, I have no problem. I felt I was protected. If [I have] no PPE, I feel no protection at all.”* (N01)

During the shortage of PPE, management had to control its use of PPE. They limited the staff and did only emergency surgeries. No elective surgery was performed in the COVID-19 hospital. They prioritized staff such as for those who were involved directly with COVID-19 patients for PPE use. The other health staffers were only provided one mask for a whole day.

To overcome the shortage of PPE, the management, with help from the health staff, took the initiatives to produce it themselves. They used garbage plastic bags as head covers, used sterilization crepe paper for temporary head and shoe covers, formed teams to sew PPE, and found a sponsor to buy materials for the PPE and supply face shields. Fortunately, this situation only lasted for a week before they got enough proper PPE supply from government and nongovernment organizations.

#### Subtheme 3.4: Spiritual and Mental Strength

Spiritual and mental strength was essential for coping with the COVID-19 pandemic. Good mindset and motivation, inner self-adjustment, religious practices, sincerity, and patient acceptance of responsibility have acted as important ways to adapt to the current situation. A good mindset has been a motivation for them to continue working in difficult situations.

“*If other people can do it, I can do it too. Okay, I said, never mind. Maybe the matron sent me to learn. You sent me here, I can do the work. I do what I can do*.” (N08)

“*We set our minds to be okay*.” (MA05)

Inner self-adjustment also made them comfortable when they were being shuffled around even though they felt that learning new responsibilities at the beginning of working in the new environment was difficult. Religious practices such as leaving everything to God after they had done their best, praying for better days, and regarding work as worship helped the health staff to accept responsibility for the job with sincerity and patience. Their job required that they put aside their fear, and this inner strength helped them handle the situation well.

#### Subtheme 3.5: Preparation for Worse Situations

Healthcare providers have prepared themselves for a possible new increase in cases. However, they hope that the public will play a role in battling this pandemic together. The public needs to adhere to new normal lifestyles like social distancing, good hand hygiene, and mask-wearing to prevent transmission and reduce cases.

The health staff has also suggested that hospital management needs to ensure adequate staff numbers so the workloads are reduced and to provide enough full PPE in case cases increase again. They hope that they will do better in the next pandemic.

### Theme 4: Future Expectations

#### Subtheme 4.1: Hope

The healthcare providers hope that COVID-19 cases will diminish in number and disappear 1 day with medicines or vaccines that will effectively cure and prevent the disease. They praised the role the government played in flattening the COVID-19 curve. They also hope that the public will be more responsible and follows the government's rules and guidelines to prevent an increase in COVID-19 cases and that their superiors in management will be more tolerant in assigning and shuffling staff.

#### Subtheme 4.2: Lessons Learned

The preparation of adequate equipment like PPE is necessary if health staffers are to be able to work efficiently with peace of mind. Hospital management needs to take care of the welfare and emotions of the reshuffled staff, listen to their voices of dissatisfaction, and take proper actions so that they feel comfortable adapting to the new working environment. During the early period of the COVID-19 pandemic, the nurses complained about swab test screening that was only available for those who had symptoms, while those who had no symptoms were not eligible for swab test screening even if they had a positive COVID-19 colleague. They also suggested that bonuses be given to backline healthcare providers working outside their departments to cover the essential tasks left by staff deployed as frontline providers. More health staffers need to be prepared so that the working hours can be shortened and workloads reduced.

#### Subtheme 4.3: Recommendations

Healthcare providers have recommended that management improve the availability of swab test screening. This screening should be eligible for the safety and tranquility of all health staffers who have exposure to COVID-19 patients or colleagues while they work in high-risk areas, even if they do not experience any symptoms. However, they understand the limitations of the costs, instruments, and facilities for swab test screening.

## Discussion

This qualitative study explored the experiences and challenges faced by frontline healthcare providers and the coping strategies they have adopted in response to the COVID-19 pandemic. Four themes were identified by ICU health staffers consisting of medical officers, medical assistants, and nurses: invaluable experiences during the pandemic, challenges faced by healthcare providers, coping strategies, and future expectations. The commitment shown by healthcare providers is very commendable. Healthcare providers are willing to fight a disease for which there is still no cure. The challenging experiences that they gathered during this pandemic might be invaluable.

The first theme of this study was invaluable experiences during the pandemic. Physicians, nurses and other medical professionals who have their responsibilities might have similar or different experiences in combating COVID-19 (29). The pandemic has impacted the healthcare working system which affected the management and working environment (30). Healthcare workers have had new experiences during changes in working practice with new roles and responsibilities during this pandemic (31) including rescheduling working hours, redeployment, and retraining. The changes also gave the psychological impacts such as stress. Stress was triggered when healthcare providers managed and treated COVID-19 patients for the first time with less knowledge about the disease and without proper preparation to handle COVID-19 cases (32). It became worse for staff with different skills and training shuffled in from different specialties, who faced problems in managing patients in the ICU (33).

These experiences also related to the second theme which was challenges faced by healthcare providers. Undeniable, healthcare providers have faced many challenges during this pandemic. The challenges came from multi-sources such as community, management, and personal thoughts. The worst part was in the initial phase of the pandemic when they had inadequate protective gear but were exposed to infected patients; then they were forced to wear uncomfortable PPE for long hours, and the health staff was short (34). There was also personal stress, including the fear of transmission of the disease to family members in vulnerable groups like older parents, parents with comorbidities, and young children (35).

Experiences in dealing with life-threatening illnesses teach them to cope with the situation (third theme) and become more resilient (35).

Obligation and responsibility toward their professions made all surveyed healthcare providers continue working under any circumstances (32). Higher resilience is also associated with an increased willingness to work as frontline healthcare providers (15). They were capable of managing the challenges related to the pandemic effectively. For example, to continue working in dangerous conditions and for their safety, they became more creative and innovative in finding solutions to overcome the shortage of PPE while waiting for the government to supply it. With support from management, the healthcare providers collaborated with public and non-governmental organizations to provide temporary PPE. The health staff were provided with proper procedures for donning and doffing PPE and guidelines for standard operating procedures. The guidelines and standard operating procedures are essential for protecting our health and preventing the transmission of the disease.

The actions taken by the government, specifically the Ministry of Health (36), and the support system implemented by management have increased healthcare providers' resilience (34). The government has allocated bonuses to show appreciation for the sacrifice of frontline healthcare providers in fighting against COIVD-19 (36). Management and the government have built a support system for their mental well-being. All health staff has access to the hotline of the support system for managing mental health (37). The public and NGOs have also provided social support for healthcare providers to enhance their resilience by providing them with food and beverages. The public and NGOs also voluntarily prepared temporary PPEs for the healthcare providers during the shortage of equipment (38).

As a future expectation theme since the healthcare workers have successfully faced the challenges of the first wave of the COVID-19 pandemic, they have learned a lesson, gained invaluable experiences, and expect the future to improve health services and enhance their resilience toward COVID-19. During this hardship, the healthcare management played their role and made efforts to combat the pandemic (15), and at the same time provide safety measures to reduce the staff's likelihood of COVID-19 infection. They built a team to control and manage the admission of COVID-19 patients and their transportation to the isolation ward with special procedures for the safety of their staff. They also rescheduled the shifts and rotated the staff to reduce the hours of exposure to infected patients and maintained the staff's physical health with a system of 2 days followed by 2 days off. However, shuffling of staff is needed to overcome the shortage due to staff deployment out of the station to serve on the front line.

Malaysia imposed MCOs in the early phase of the pandemic to flatten the curve of cases (39). However, this partial lockdown negatively impacted the economy, and some members of the public have experienced financial difficulties (36). The orders recommended that people “stay at home,” wear a mask in public, keep social distance, and practice hand hygiene to protect themselves from infection. Healthcare providers advised the public to follow the guidelines and standard operating procedures for their benefit and to reduce the number of cases. Healthcare providers, especially nurses, hope that management will increase the number of staffers and prepare the equipment well, especially the PPEs, to provide better services for the public's health and battle more cases of COVID-19.

### Limitations

This study only involved healthcare providers who worked in the ICU during the COVID-19 pandemic. Different study settings might yield different results. The choice of participants may have introduced study bias, as the choice of respondents was based on the willingness and availability of the respondents.

## Conclusion

The healthcare providers showed that facing the challenging experiences of the COVID-19 pandemic increased their resilience over what it had been before the pandemic. They managed to cope and adapt well to the new lifestyle and new working environment and do well in their jobs. Less perceived negative psychological impact shows that healthcare providers' mental health was good even during the pandemic. Good mental health protects medical professionals from suffering in combating the pandemic and saves lives. Hopefully, this study may give a critical information for future psychological interventions among healthcare professionals especially during disease pandemics.

## Data Availability Statement

The raw data supporting the conclusions of this article will be made available by the authors, without undue reservation.

## Ethics Statement

The studies involving human participants were reviewed and approved by the Universiti Sains Malaysia Human Research Ethics Committee (USM/JEPeM/COVID19-10) the Ministry of Health Medical Research Ethics Committee (NMRR-20-703-54576). The patients/participants provided their written informed consent to participate in this study.

## Author Contributions

MN: conceptualization, methodology, data curation, visualization, supervision, and correspondence. RC: software, formal analysis, and writing—original draft preparation. RC and MN: validation. MN and YA: writing—review and editing. All authors have read and agreed to the published version of the manuscript.

## Conflict of Interest

The authors declare that the research was conducted in the absence of any commercial or financial relationships that could be construed as a potential conflict of interest.

## Publisher's Note

All claims expressed in this article are solely those of the authors and do not necessarily represent those of their affiliated organizations, or those of the publisher, the editors and the reviewers. Any product that may be evaluated in this article, or claim that may be made by its manufacturer, is not guaranteed or endorsed by the publisher.
